# Altered frontal electroencephalography as a potential correlate of acute dissociation in dissociative disorders: novel findings from a mirror confrontation study

**DOI:** 10.1192/bjo.2022.593

**Published:** 2022-11-10

**Authors:** Eva Schäflein, Yoki Linn Mertens, Nena Lejko, Sarah Beutler, Heribert Sattel, Martin Sack

**Affiliations:** Department of Psychosomatic Medicine and Psychotherapy, University Hospital of Erlangen, Friedrich-Alexander University Erlangen-Nürnberg, Germany; and Department of Psychosomatic Medicine and Psychotherapy, Hospital rechts der Isar of the Technical University of Munich, Germany; Department of Clinical Psychology and Experimental Psychopathology, University of Groningen, The Netherlands; Cognitive Neuroscience Center, Department of Biomedical Sciences of Cells & Systems, University Medical Center Groningen, University of Groningen, The Netherlands; Department of Psychotherapy and Psychosomatic Medicine, Medical Faculty, Technical University of Dresden, Germany; Department of Psychosomatic Medicine and Psychotherapy, Hospital rechts der Isar of the Technical University of Munich, Germany

**Keywords:** Dissociative disorders, electroencephalography, post-traumatic stress disorder, mirror, self-perception

## Abstract

People suffering from chronic dissociation often experience stress and detachment during self-perception. We tested 18 people with dissociative disorders not otherwise specified (DDNOS; compared with a matched sample of 18 healthy controls) undergoing a stress-inducing facial mirror confrontation paradigm, and measured acute dissociation and frontal electroencephalography (measured with a four-channel system) per experimental condition (e.g. confrontation with negative cognition). Linear mixed models indicated a significant group×time×condition effect, with DDNOS group depicting less electroencephalography power than healthy controls at the beginning of mirror confrontation combined with negative and positive cognition. This discrepancy – most prominent in the negative condition – diminished in the second minute. Correlational analyses depicted a positive association between initial electroencephalography power and acute dissociation in the DDNOS group. These preliminary findings may indicate altered neural processing in DDNOS, but require further investigation with more precise electroencephalography measures.

Dissociation is difficult to assess because of heterogeneity in conceptualisations and diagnostics, often limited by responder biases or deficient interoceptive abilities of patients, highlighting the need to identify reliable biomarkers. Neurobiological models^1,2^ propose elevated activity of the medial-frontal brain regions as a neurofunctional biomarker of pathological dissociation, resulting in blunted emotional and sensory experiencing. Neuroimaging research suggests a relationship between (acute) dissociation and the activation of prefrontal brain areas during stress-inducing paradigms.^3–6^ Whereas neuroimaging techniques present good spatial resolution, non-invasive neurophysiological measures such as electroencephalography (EEG) are a viable option to extract brain–behaviour correlates with high temporal resolution and better clinical applicability.^7^ To date, EEG research on pathological dissociation remains scarce (see Roydeva and Reinders^8^); singular findings in patients with dissociative disorder point to blunted EEG connectivity^9^ and decreased relative theta magnitude in bilateral temporal cortices associated with increased acute dissociation.^10^ Schlumpf and colleagues^11,12^ found hypoconnectivity in emotion regulatory networks (in the beta frequency band) and resting-state networks (in the theta and alpha frequency band) in patients with complex dissociative disorder who were pre-treatment. Additionally, a case study by Sartorius and Schmahl^13^ used bispectral index monitoring and found that the bispectral index, a measure of consciousness, was lower during a dissociative episode in a patient with borderline personality disorder.

## Study aim

The current study's aim was to examine neural processing underlying acute dissociation by monitoring EEG in people with dissociative disorder not otherwise specified (DDNOS type 1), during a facial mirror confrontation task. Self-perception has previously been shown to elicit dissociative detachment sensations (i.e. depersonalisation and derealisation) in DDNOS.^14^ Thus, we expected that the DDNOS group (compared with a group of healthy controls) would depict increased frontal EEG total power when undergoing the experimental procedure. Additionally, we wanted to explore if frontal EEG total power, assessed via a four-channel EEG montage called BIS-VISTA, correlated with acute dissociation. BIS-VISTA is a simple, easy-to-use, four-electrode EEG set-up that is normally used to monitor people under anaesthesia. However, it can also be used to measure EEG during wakefulness. Although the system is named after the bispectral index – one of the measures used to monitor the depth of anaesthesia – it also measures total power, which we employed in our study.

## Method

### Participants and procedure

The DDNOS and healthy control groups included 18 persons each (17 female). Mean age was 41.7 (s.d. = 8.3) years in DDNOS and 41.1 (s.d. = 10.0) years in healthy controls; groups did not statistically differ in age (*t*(34) = 0.18; *P* = 0.857). Diagnosis of DDNOS (type 1) was assessed by the Mini-Structured Clinical Interview for DSM-IV – Dissociative Disorders^15^ (short version; cut-off 10 out of 15). Trait dissociation was measured by the Dissociative Experiences Scale (DES).^16^ In distinct phases of 2 min each, participants first looked at their own face in a mirror without any accompanying cognition (mirror confrontation, MConly), and then in combination with either an instructed negative (MCneg) or positive (MCpos) cognition. For the negative condition, participants chose the most disturbing negative cognition about themselves from Shapiro's eye movement desensitisation and reprocessing manual (e.g. themes of guilt, danger or self-worth).^17^ EEG was measured throughout the experiment. Participants reported severity of acute dissociation with the dissociation subscale of the Responses to Script-Driven Imagery Scale (RSDI)^18^ after each phase. For a detailed description of the sample and procedures, see Schäflein et al.^14^ Schäflein et al^14,19^ and Schäflein^20^ are partially based on the same data-set.

### Total EEG power

We measured total EEG power by means of a BIS-VISTA Bilateral Monitoring System (a bispectral index monitor; Aspect Medical Systems, Massachusetts, USA). EEG was recorded with five Ag/AgCl electrodes (Covidien, Massachusetts, USA) attached to the forehead (10/20 system locations: Fp1, Fp2, A1, A2, plus a central reference electrode). Data were sampled with a frequency of 256 Hz and subjected to analogue bandpass filtering of 0.3–70 Hz. Data were excluded from analysis if electrode impedances were >7.5 kOhm or if contamination by visible gross artifacts or a signal quality index <80% were detected. For each second of the recording, the BIS-VISTA Monitoring System automatically calculated total EEG power in the 0.5–30 Hz frequency range. Values from the two hemispheres were averaged to obtain bilateral frontal total EEG power.

### Statistical analysis

**Fig. 1:**
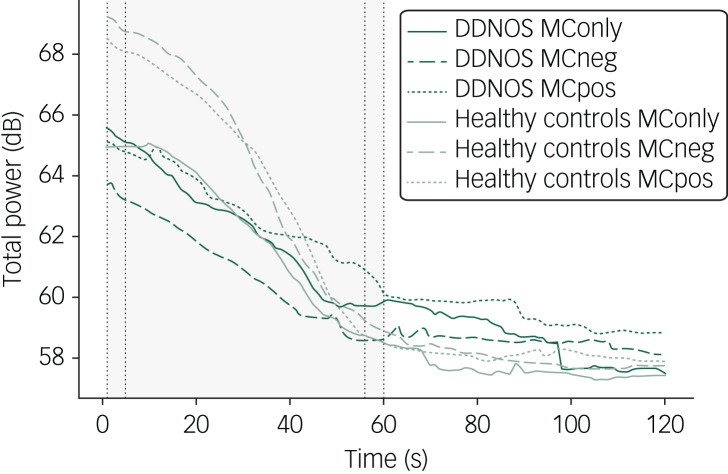
Time course of total power during mirror confrontation. Total frontal electroencephalography power in dB, plotted against time for the entire duration of mirror confrontation. The first minute of the signal used for analysis is highlighted in grey, and the first and last 5-s segments are denoted by vertical dotted lines. DDNOS, people with dissociative disorder not otherwise specified; MConly, mirror confrontation only; MCneg, mirror confrontation with negative cognition; MCpos, mirror confrontation with positive cognition.

The analysis was confined to the first minute, as visualising the data revealed the completion of the adaptive processes within this time (Fig. 1). The time course of total power was divided into 10-s intervals, with the first and last interval only spanning 5 s each, heuristically respecting early signal attenuation.^21^ Linear mixed models were applied with participants and conditions as random, and interval and group allocation as fixed effects. Age and antidepressant use were added as covariates. To estimate the full model, a group×condition×interval interaction was determined. For group comparisons within each condition, a group×condition interaction was estimated.

For baseline comparisons of EEG power between groups, independent *t*-tests were applied. Because of the multiple statistical comparisons (three conditions at two different intervals), alpha was set to 0.05/6, resulting in an alpha of 0.008. Total EEG power in the first interval was correlated with acute and trait dissociation (RSDI and DES) within the DDNOS group only, as the healthy control group did not experience any acute dissociation.

The authors confirm that all procedures contributing to this work comply with the ethical standards of the relevant national and institutional committees on human experimentation and with the Helsinki Declaration of 1975, as revised in 2008. All procedures involving human patients were approved by the Ethics Committee of the Technical University of Munich, Germany (approval number 1/14 S). Written informed consent was obtained from all participants.

## Results

On average, the DDNOS group reported experiencing trait dissociation (DES) >27% of the time (mean 27.86, s.d. = 9.28), whereas the healthy control group did not report any. The full linear mixed model controlling for age and psychoactive drugs revealed a statistically significant group×condition×interval interaction, indicating that EEG power during the first minute of mirror confrontation followed different trajectories for people with DDNOS and healthy controls (*F*(2, 705.3) = 8.68, *P* = 0.001). None of the control variables exerted a significant effect on total power. The individual participants’ contributions accounted for 49.6% of the observed variance, whereas the proportion explained by the experimental condition was negligible (3.4%).

In the MConly condition, total EEG power did not differ between the two groups in either the first interval (*t*(34) = 0.23, *P* = 0.823) or the last interval (*t*(28.7) = 1.04, *P* = 0.307). In the MCneg condition, total EEG power was initially elevated in healthy controls, but not in people with DDNOS (*t*(34) = −3.57, *P* = 0.001). In the last interval, total power for healthy controls decreased to a point where the between-group difference was no longer significant (*t*(34) = −0.06, *P* = 0.950). A similar but less pronounced effect was observed for the MCpos condition (first interval: *t*(27.8) = −2.02, *P* = 0.054; last interval: *t*(34) = −1.40, *P* = 0.171). The linear mixed model analysing the experimental conditions separately revealed a group×interval interaction for the MCneg and MCpos conditions (*F*(1, 208) = 39.22, *P* = 0.001 and *F*(1, 214) = 32.60, *P* = 0.001, respectively), but not for MConly (*F*(1, 214) = 1.63, *P* = 0.204).

Within people with DDNOS, trait and acute dissociation were moderately correlated (*r*(18) = −0.40, *P* = 0.099). Higher EEG power during the first interval of the MCneg condition was related positively to more severe acute dissociation (*r*(18) = 0.51, *P* = 0.029), but this was not the case for MConly (*r*(18) = −0.182, *P* = 0.471) or the MCpos condition (*r*(18) = −0.18, *P* = 0.464). Trait dissociation did not correlate significantly with initial total EEG power in any of the conditions.

## Discussion

The current study sought to identify biobehavioural correlates of acute dissociation by measuring frontal EEG in patients with DDNOS following a stress-inducing facial mirror confrontation paradigm. Compared with healthy controls, people with DDNOS had lower total EEG power when mirror confrontation was paired with a negative or positive cognition, opposite to the hypothesised direction. Interestingly, experimentally elicited acute dissociation in people with DDNOS correlated positively with total EEG power at the beginning of the negative condition, although this was statistically non-significant after considering a correction for multiple testing. Notably, this association was not present for trait dissociation, suggesting that this neural correlate is specific for acute dissociation experienced during the self-perception paradigm. Altogether, the present findings appear contradictory. Between groups, the healthy control group displayed higher frontal EEG power compared with the clinical group in the negative condition; thus, lower frontal EEG power appears a marker for pathological dissociation. This group difference is difficult to interpret as the experiment did not elicit any meaningful stress or dissociation levels in the healthy control group (see Schäflein et al^14^), and therefore may reflect another component (e.g. attention) that is not related to the clinical expression, but more to the task itself. Within-group elevated frontal EEG power was associated with increased acute dissociation in the DDNOS group. Conceivably, this finding would align with previous research suggesting elevated activity in prefrontal regions are potential markers of stress-induced acute dissociation,^3–6^ concurrent with a blunted psychophysiological response.^14,18^

Several limitations of this study should be acknowledged. The EEG was recorded with a four-electrode system that is normally used to monitor patients undergoing anaesthesia. Although this decision was made to avoid additionally burdening the already anxious participants with DDNOS by placing more electrodes, it also introduced considerable limitations. First, the output of the EEG monitoring system used in this study was limited to precalculated parameters. Since the BIS-VISTA system does not provide the precise spectral data, we had to use power in the 0.5–30 Hz frequency band as the output measure that was the most appropriate for use in awake, conscious individuals. However, although EEG power in narrower frequency bands has well-established behavioural correlates, the power of a wide band cannot be easily interpreted. The frequency band analysed here includes the alpha band (higher power equals cortical suppression) as well as delta, theta and beta bands (higher power equals cortical activation).^22,23^ We are therefore unable to draw conclusions about whether an increase in total power reflects cortical activation or suppression. Second, the EEG system only provided values for every second, whereas EEG is usually measured in milliseconds. Thus, the normally high temporal resolution of EEG has been lost, and some between- and within-group differences could have been masked by averaging. Third, given the poor spatial resolution of EEG, we cannot ascertain whether the activity measured at the front of the scalp truly originates from frontal brain regions. Because EEG was only recorded at two frontal sites and the BIS-VISTA system does not provide precise spectral data, we cannot check whether our results are unique to the activity recorded at frontal sites, or make use of source localisation methods to investigate where the measured activity likely originated from. Furthermore, the RSDI scale only covers dissociative detachment symptoms (depersonalisation and derealisation), which also occur in several non-dissociative disorders. Thus, we do not know if any fragmentation symptoms (e.g. amnesia, identity alteration) of dissociative disorders and thus severe pathological dissociative symptoms occurred during the self-perception task.

Given the above limitations, our findings should be interpreted with extreme caution; however, they may be seen as one more step toward investigating the neural mechanisms of dissociation and self-perception. To further investigate the potential of EEG as a biomarker of dissociation, future studies should utilise traditional, higher-density electrode configurations to investigate dissociation-related activity with higher frequency and temporal and spatial resolution, as well as a state scale of dissociation that measures the whole range of dissociative symptoms.

## Data Availability

The data that support the findings of this study are available from the corresponding author, E.S., upon reasonable request.

## Supplementary material

For supplementary material accompanying this paper visit http://doi.org/10.1192/bjo.2022.593. click here to view supplementary material
