# Supplementary material for: Altered frontal electroencephalography as a potential correlate of acute dissociation in dissociative disorders: novel findings from a mirror confrontation study
Source: BJPsych Open. 2022 Nov 10;8(6):e196. doi: 10.1192/bjo.2022.593 (PMC9707509; doi:10.1192/bjo.2022.593)
Supplement: Supplementary file 1 [file S2056472422005932sup001.docx]

Supplementary Material

Supplementary Table 1. Correlations TOTPOW - psychophysiological parameters

| Group x condition. first 10 s interval | |  |  |
| --- | --- | --- | --- |
| Patients (N=18) | | IBI (ms) | RMSSDln |
| MC only | correlation (Pearson) | -.025 | .322 |
|  | Sig. (2-sided) | .921 | .192 |
| MC neg | correlation (Pearson) | -.392 | .004 |
|  | Sig. (2-sided) | .108 | .988 |
| MC pos | correlation (Pearson) | -.093 | -.051 |
|  | Sig. (2-sided) | .712 | .841 |
| Controls (N=18) | | IBI (ms) | RMSSDln |
| MC only | correlation (Pearson) | -.114 | -.364 |
|  | Sig. (2-sided) | .654 | .138 |
| MC neg | correlation (Pearson) | -.101 | -.148 |
|  | Sig. (2-sided) | .690 | .557 |
| MC pos | correlation (Pearson) | .133 | .239 |
|  | Sig. (2-sided) | .600 | .339 |

*Note.* IBI = interbeat interval; MC only = mirror confrontation without any cognition; MC neg = mirror confrontation with negative cognition, MC pos = mirror confrontation with positive cognition; RMSSDln = natural logarithm of Root Mean Square of Successive Differences.

Supplementary Table 2. TOTPOW left vs. right hemispheres

| Condition = MC only | | Hemi both | Hemi left | Hemi right | Hemi difference (left – right) |
| --- | --- | --- | --- | --- | --- |
| Patients | M | 62.2852 | 62.4428 | 62.1541 | .2463 |
|  | SD | 5.14715 | 5.27914 | 5.12978 | 1.81407 |
| Controls | M | 62.3073 | 62.3976 | 62.2171 | .1538 |
|  | SD | 4.00779 | 4.29253 | 3.94218 | 2.01842 |
| Condition = MC neg | |  |  |  |  |
| Patients | M | 60.9014 | 61.0430 | 60.7622 | .3159 |
|  | SD | 4.63306 | 4.90402 | 4.57476 | 2.09771 |
| Controls | M | 64.2177 | 64.3317 | 64.1112 | .1957 |
|  | SD | 5.67896 | 5.92409 | 5.63097 | 2.13454 |
| Condition = MC pos | |  |  |  |  |
| Patients | M | 62.7680 | 62.7933 | 62.7430 | .1128 |
|  | SD | 5.18143 | 5.13012 | 5.32418 | 1.49763 |
| Controls | M | 63.9534 | 63.8949 | 64.0118 | -.1689 |
|  | SD | 4.65443 | 4.66525 | 4.84991 | 2.09955 |

*Note*. M = mean; MC only = mirror confrontation without any cognition; MC neg = mirror confrontation with negative cognition, MC pos = mirror confrontation with positive cognition; SD = standard deviation.

Figure S1. Time course of total power in the left hemisphere during mirror confrontation


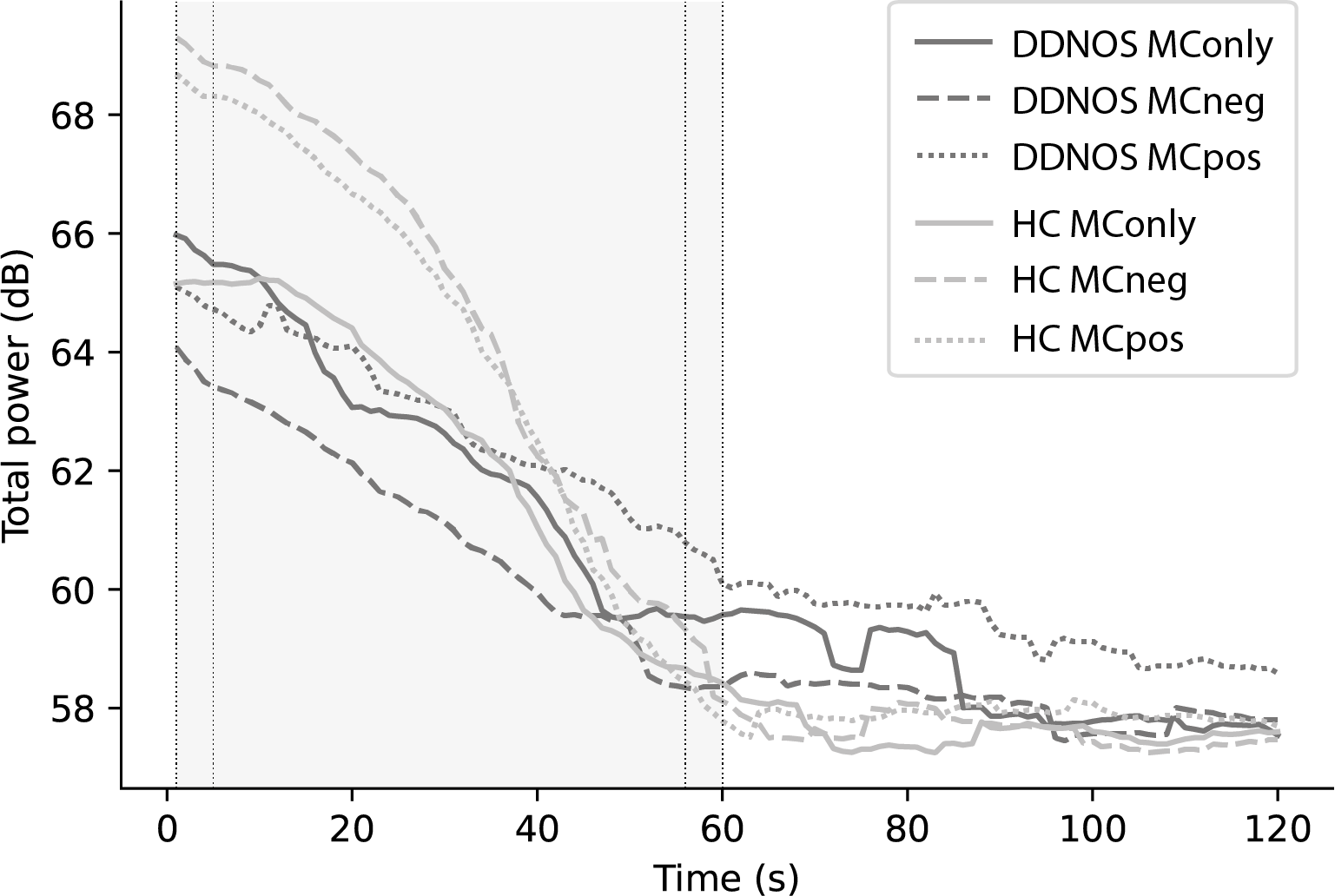


*Note.* Total frontal EEG power in dB plotted against time for the entire duration of mirror confrontation (MC). The first minute of the signal used for analysis is highlighted in grey, and the first and last 5 s segments are denoted by vertical dotted lines. DDNOS = people with dissociative disorder not otherwise specified; HC = healthy controls; MConly = MC without any cognition; MCneg = MC with negative cognition, MCpos = MC with positive cognition.

Figure S2. Time course of total power in the right hemisphere during mirror confrontation


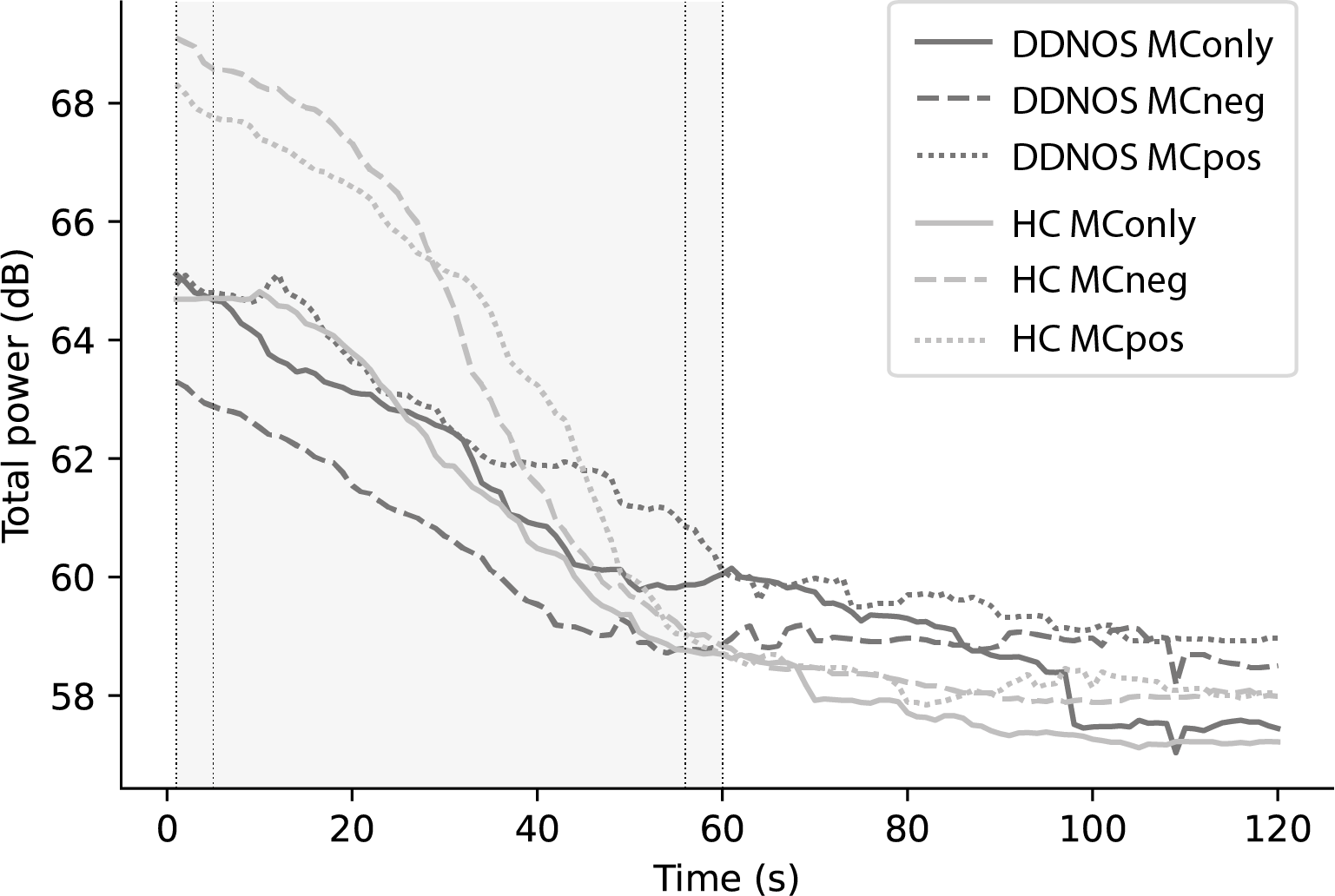


*Note.* Total frontal EEG power in dB plotted against time for the entire duration of mirror confrontation (MC). The first minute of the signal used for analysis is highlighted in grey, and the first and last 5 s segments are denoted by vertical dotted lines. DDNOS = people with dissociative disorder not otherwise specified; HC = healthy controls; MConly = MC without any cognition; MCneg = MC with negative cognition, MCpos = MC with positive cognition.

Supplementary Table 3. Linear mixed models

| Fixed effects | Hemi both | | | Hemi left | | | Hemi right | | | Hemi difference  (Hemi left – Hemi right) | | |
| --- | --- | --- | --- | --- | --- | --- | --- | --- | --- | --- | --- | --- |
| MC only | df | F-value | Sig. | df | F-value | Sig. | df | F-value | Sig. | df | F-value | Sig. |
| Constant | 43.729 | 10290.9 | .000 | 45.884 | 10032.1 | .000 | 42.848 | 9827.27 | .000 | 68.980 | 8.809 | .004 |
| Time | 206.993 | 190.335 | .000 | 208.000 | 186.634 | .000 | 206.99 | 165.177 | .000 | 205.167 | 14.890 | .000 |
| Group | 43.729 | .411 | .525 | 45.884 | .282 | .598 | 42.848 | .534 | .469 | 68.980 | .505 | .480 |
| Group * Time | 206.993 | 3.105 | .080 | 208.000 | 2.075 | .151 | 206.99 | 3.994 | .047 | 205.167 | .872 | .352 |
| MC neg |  |  |  |  |  |  |  |  |  |  |  |  |
| Constant | 43.862 | 9219.30 | .000 | 42.753 | 8032.13 | .000 | 46.236 | 9687.94 | .000 | 55.552 | 2.360 | .130 |
| Time | 206.141 | 227.077 | .000 | 206.141 | 222.908 | .000 | 206.16 | 196.621 | .000 | 205.571 | 2.060 | .153 |
| Group | 43.862 | 21.344 | .000 | 42.753 | 16.589 | .000 | 46.236 | 24.919 | .000 | 55.552 | 1.715 | .196 |
| Group * Time | 206.141 | 35.707 | .000 | 206.141 | 27.349 | .000 | 206.16 | 38.629 | .000 | 205.571 | 5.223 | .023 |
| MC pos |  |  |  |  |  |  |  |  |  |  |  |  |
| Constant | 45.693 | 10650.0 | .000 | 45.766 | 11213.0 | .000 | 46.377 | 9500.12 | .000 | 61.989 | 1.223 | .273 |
| Time | 208 | 222.119 | .000 | 208.000 | 247.063 | .000 | 208.00 | 178.668 | .000 | 207.149 | 5.356 | .022 |
| Group | 45.693 | 9.972 | .003 | 45.766 | 11.834 | .001 | 46.377 | 7.831 | .007 | 61.989 | .302 | .585 |
| Group * Time | 208 | 33.141 | .000 | 208.000 | 43.247 | .000 | 208.00 | 22.159 | .000 | 207.149 | 4.299 | .039 |

*Note*. MConly = mirror confrontation without any cognition; MC neg = mirror confrontation with negative cognition, MC pos = mirror confrontation with positive cognition.
